# ALOX5 Expression and Pathomics Features Reveal New Insights Into Lung Adenocarcinoma Prognosis: Model Construction and Functional Validation

**DOI:** 10.1155/humu/3303601

**Published:** 2026-03-09

**Authors:** Peihong Hu, Chun Huang, Xin Liu, Qiang Li, Run Xiang, Xu Liang

**Affiliations:** ^1^ Department of Thoracic Surgery, Sichuan Clinical Research Center for Cancer, Sichuan Cancer Hospital & Institute, Sichuan Cancer Center, School of Medicine, University of Electronic Science and Technology of China, Chengdu, China, uestc.edu.cn; ^2^ School of Medicine, University of Electronic Science and Technology of China, Chengdu, China, uestc.edu.cn; ^3^ Department of Thoracic Surgery, Sichuan Clinical Research Center for Cancer, Sichuan Cancer Hospital & Institute, Sichuan Cancer Center, University of Electronic Science and Technology of China, Chengdu, China, uestc.edu.cn; ^4^ Department of Radiology, Sichuan Clinical Research Center for Cancer, Sichuan Cancer Hospital & Institute, Sichuan Cancer Center, University of Electronic Science and Technology of China, Chengdu, China, uestc.edu.cn

**Keywords:** arachidonic acid 5-lipoxygenase, lung adenocarcinoma, pathological mechanism, pathomics, prognosis prediction

## Abstract

**Background:**

Arachidonic acid 5‐lipoxygenase (ALOX5) is a biomarker of lung adenocarcinoma (LUAD). This research seeks to establish a prognostic model for LUAD by examining ALOX5 expression.

**Methods:**

The Cancer Genome Atlas database provided the pathological images and transcriptome data. To determine the prognostic value of ALOX5, survival analysis and Cox regression (univariate and multivariate) were conducted, along with subgroup analysis and interaction tests. The OTSU algorithm and the PyRadiomics package were used to segment the pathological images of patients with LUAD and extract pathological features. The gradient‐enhanced model algorithm was used to construct the pathological omics model. The prognostic value of pathomics mechanism analysis was confirmed using the pathomics score (PS) output from the model. Cell experiments were used to verify gene function.

**Results:**

A total of 327 samples and seven best pathological features were included in the analysis. In LUAD, elevated levels of ALOX5 and PS were associated with improved overall survival. The gradient boosting machine (GBM) pathomics model demonstrated strong predictive performance and clinical applicability, achieving an area under the curve (AUC) of 0.786 in the training set (*n* = 230) and 0.741 in the validation set (*n* = 97). According to the model, samples with elevated ALOX5 expression exhibited higher PS values. Moreover, macrophage infiltration was significantly increased in groups with high PS expression. Gene set enrichment analysis (GSEA) indicated that genes differentially expressed between PS subgroups were involved in apoptosis and inflammatory‐response pathways. Apoptosis‐related genes were positively correlated with PS values (*p* < 0.001), and 63 hub genes associated with the inflammatory response were enriched in cytokine‐mediated signaling pathways. In vitro experiments showed that ALOX5 knockdown in lung cancer cells enhanced tumor cell proliferation and migration.

**Conclusions:**

There was a strong link between ALOX5 expression levels and OS in LUAD patients. A pathomics‐based model can effectively predict the expression level of ALOX5; as a result, LUAD patients′ prognoses can be predicted.

## 1. Introduction

Cancer‐related deaths are most commonly caused by lung cancer [1]. Among primary classifications of pulmonary malignancies, non–small cell lung cancer constitutes approximately 85% of diagnosed instances, whereas small cell carcinomas represent the minority proportion at 15% [2]. Approximately two‐fifths of non–small cell lung carcinoma diagnoses are attributable to the adenocarcinoma histological subtype [3]. Patients with advanced LUAD have a poor prognosis, similar to that for most solid tumors, and identifying a high‐risk classification of poor prognosis is crucial. Traditional prognostic indicators for LUAD, such as clinicopathological features, laboratory diagnostics, and computed tomography images, are inadequate for early diagnosis and treatment. Many researchers are attempting to discover noninvasive and effective prognostic markers that can be used to diagnose lung cancer early and provide a longer life expectancy. For example, Xu et al. [4] discovered that heat shock protein 90 *β* can be a potential prognostic tool for LUAD. Wang et al. [5] observed that the expression of dipeptidase‐2 is associated with cisplatin sensitivity and may be useful as a prognostic indicator for LUAD. However, these biomarkers cannot predict the prognosis efficiently; therefore, studies are being conducted to identify suitable prognostic biomarkers for clinical diagnosis and treatment.

Arachidonic acid 5‐lipoxygenase (LOX) belongs to the LOX family. Research indicates that ALOX5‐encoded 5‐LOX is pivotal in tumor progression. Liu et al. [6] found that ALOX5 deficiency promotes bladder cancer progression by enabling escape from ferroptosis. Wang et al. [7] reported that ALOX5 cut in melanoma cells correlates with positive patient prognosis. The prognosis of ALOX5 levels and low‐grade gliomas was significantly correlated, according to Liu et al. [8]. These studies have confirmed the importance of ALOX5 in tumor prognosis research, and thus, we conducted this study on ALOX5. The assessment of ALOX5 expression levels primarily depends on methods such as peripheral blood cytokine detection, which, despite being real‐time, is costly and does not capture the tumor′s intrinsic nature. Alternatively, mRNA levels can be measured through quantitative polymerase chain reaction and RNA‐seq, while protein levels are assessed using western blotting (WB) and flow cytometry, both requiring fresh tissue samples. Sample collection is difficult, and the detection is influenced by the technicians performing the sample collection and the types of antibodies involved; detection using paraffin‐embedded tissue samples (immunohistochemistry [IHC], fluorescence analysis, and high‐throughput sequencing) has shortcomings, such as expensive antibodies and varying technical skills of the technicians. The advantages of hematoxylin and eosin (H&E) staining are evident in clinical practice.

The integration of artificial intelligence–driven diagnostic tools into histopathological workflows is revolutionizing traditional tissue analysis methodologies, reshaping clinical decision‐making paradigms. It is employed for quantitative pathological diagnosis, molecular expression analysis, and disease prognosis evaluation [9–11]. Many applications of pathomics‐based methods in the analysis of clinical disease prognosis exist. For example, according to Chen et al. [12], a prognosis model using pathological features can predict the outcomes of patients with gastric cancer.

Considering the aforementioned factors, we attempted to predict ALOX5 expression levels in lung adenocarcinoma tissues, which was integrated with bioinformatic analysis and the help of a pathomics‐based approach to explore the possible mechanistic understanding of molecules influencing the results of the pathomics analysis.

## 2. Materials and Methods

### 2.1. Sources for Images and Data

**Figure 1:**
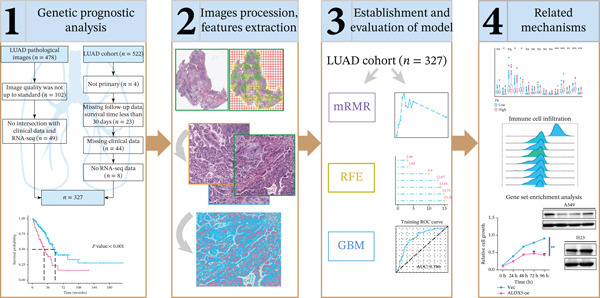
The data analysis workflow. To begin with, we identified the patients to be included in the study based on the inclusion criteria and conducted survival analysis. Second, we conducted histopathological image processing and feature extraction. Subsequently, we established a pathomics‐based predictive model and finally used bioinformatic methods to analyze pathways and used cell experiments to verify gene function. LUAD, lung adenocarcinoma; mRMR, minimum‐redundancy‐maximum‐relevance; RFE, recursive feature elimination; GBM, gradient boosting machine.

Pathological images were sourced from the Cancer Genome Atlas (TCGA) database (https://tcga-data.nci.nih.gov/tcga/). Our RNA‐seq dataset (including clinical and follow‐up data) was retrieved from TCGA database (https://portal.gdc.cancer.gov). Exclusion criteria encompassed nonprimary LUAD, absence of follow‐up data, survival time under 30 days, lack of clinical data, and samples missing RNA‐seq information. Poor‐quality TCGA‐LUAD pathological images were eliminated, and intersecting samples were screened using clinical and RNA‐seq data. The study was exempt from ethical review as it utilized data from public databases. Figure 1 illustrates the basic process of our analysis.

### 2.2. Evaluation of ALOX5′s Prognostic Significance

We downloaded and organized RNA‐seq data for the STAR process of TCGA‐LUAD project. As part of the visualization, the R package “ggplot2” was used. The “survminer” package was employed to ascertain the cutoff value for gene expression levels of ALOX5; there were two groups of patients: with high and low expression. The “forestplot” and “survival” packages were used for univariate and multivariate Cox regression analyses. Exploratory subgroup analyses examined the impact of ALOX5 expression levels (high vs. low) on patient diagnosis across various covariate subgroups.

### 2.3. Screening of Pathological Features and Model Construction

Pathological images of H&E‐stained tissues, preserved in formalin and paraffin, were provided in ScanScope Virtual Slide (SVS) format with magnifications of up to 20× or 40×. The OTSU algorithm (https://opencv.org/) was used to determine the tissue area [13] using pathological sections. We performed segmentation at 40× magnification on 1024 × 1024‐pixel image tiles, while 20× magnification was used for 512 × 512‐pixel tiles, which were subsequently upsampled to 1024 × 1024 pixels. The pathologist methodically evaluated the image samples and discarded those with significant quality issues, following strict criteria such as contamination, blurred details, or over 50% vacant space. From each diseased pathological image, we chose 10 subimages at random for further study [14]. The subimages underwent standardization, and features were extracted utilizing the PyRadiomics software (https://pyradiomics.readthedocs.io/en/latest/). We chose samples that included comprehensive pathology pictures, gene matrices, and clinical data. A ratio of 7:3 was employed to randomly allocate the samples into training and validation sets. The training cohort′s diagnostic parameters underwent normalization using *z*‐score transformation during preprocessing. The normalization of the validation cohort was performed by employing statistical parameters (mean and standard deviation values) derived from the training datasets. An analysis of clinical variable differences between groups across the datasets was performed. The optimal feature subset was filtered using recursive feature elimination (RFE) and minimum‐redundancy‐maximum‐relevance (mRMR) algorithms. We selected the following features to build the model: logarithm_glrlm_GrayLevelNonUniformity, log_sigma_5_0_mm_3D_gldm_DependenceEntropy, lbp_2D_glrlm_GrayLevelVariance, log_sigma_5_0_mm_3D_glcm_InverseVariance, original_firstorder_Mean, exponential_glrlm_LongRunLowGrayLevelEmphasis, and gradient_firstorder_Kurtosis. A model was constructed for the training set using the gradient boosting machine (GBM) algorithm.

### 2.4. Evaluation of Pathomic Models

We assessed the overall performance of image ensemble‐based predictive models using Brier scores, evaluated the calibration of the pathomics‐based predictive models by drawing calibration curves and performing Hosmer–Lemeshow goodness‐of‐fit tests, and demonstrated the clinical benefits of the pathomics‐based model using decision curve analysis (DCA).

### 2.5. Evaluation of the Prognostic Significance of the Pathomics Score (PS)

A pathomics‐based model was employed to generate the PS for the intersecting samples. The PS and clinical variables were analyzed collectively, with the survminer package determining the optimal prognostic cutoff to categorize patients into two groups. Survival outcomes across these subgroups were then compared using Kaplan–Meier methodology.

### 2.6. Pathological Mechanism Analysis

The status of immune cell infiltration for each LUAD sample was assessed with the help of the CIBERSORTx database (https://cibersortx.stanford.edu/) by submitting the gene expression matrix from each sample. Gene set enrichment analysis (GSEA) was performed using the Kyoto Encyclopedia of Genes and Genomes (KEGG) and Hallmark gene sets to explore the molecular basis of ALOX5 gene differential expression between high‐ and low‐expression groups, with the Top 20 pathways visualized. The association between PS and apoptotic gene expression was examined using Spearman′s correlation analysis. Weighted gene coexpression network analysis (WGCNA) identified prognostically relevant inflammatory response genes by generating a similarity matrix from their expression data. Hub genes within key modules were selected for Gene Ontology (GO) and KEGG enrichment analyses.

### 2.7. Cell Line and Cell Culture

Eight NSCLC cells (H1975, H23, A549, PC9, H1299, H2170, HCC827, and H460) and Human Bronchial Epithelial Cells (HBE) were acquired from Guangzhou Cellcook Biotech Co. Ltd., China. NSCLC cells were maintained in Dulbecco′s Modified Eagle Medium (Cat. C11995500BT; Gibco; United States) supplemented with 10% fetal bovine serum (Cat. FSP500; Excell; Uruguay) and 100 U/mL penicillin–streptomycin (Cat. CB010; Yamei Bio; China). The cell incubator was maintained at 37°C with 5% carbon dioxide concentration.

### 2.8. Stable ALOX5 Overexpression and Knockdown Cell Lines

The ALOX5 lentiviral overexpression vector (Cat. LV‐ALOX5 27007‐1) and the empty vector (Cat. LVCON 335), the shRNA expression vector targeting ALOX5 (Cat. LV‐ALOX5‐RNAi 134093‐1; 134094‐1; 134095‐1), and the scrambled shRNA nontarget control (Cat. CON313(hU6‐MCS‐CBh‐gcGFP‐IRES‐puromycin)) were procured from Genechem Syngentech Corporation (China).

### 2.9. WB

Protein concentration was measured using a bicinchoninic acid (BCA) kit (Cat. P0010; Beyotime; China). Sodium dodecyl sulfate polyacrylamide gel electrophoresis (SDS‐PAGE) was performed at a concentration of 10%. Proteins were transferred to PVDF membranes and blocked with 5% skim milk. Membranes were treated with primary antibodies (Cat. 1015314‐23; 1:1000; Abcam; United States) overnight at 4°C. Treatment with secondary antibodies (Cat. AS014; 1:3000; ABclonal, China) was incubated at room temperature for 1 h, followed by luminescent solution development (Cat. WBKLS0500; Millipore; Germany).

### 2.10. Quantitative Real‐Time Polymerase Chain Reaction (qRT‐PCR)

ABScript II RT Master Mix for qPCR with DNA Remover Kit (Cat. RK20429; ABclonal; China) was used for the reverse transcription process. 2X Universal SYBR (Synergetic Binding Reagent) Green Fast qPCR Mix Kit (Cat. RK21203; ABclonal; China) was used for the qPCR process. The primers were designed as follows:

ALOX5: forward: 5 ^′^‐ACAAGCCCTTCTACAACGACT‐3 ^′^; reverse: 5 ^′^‐AGCTGGATCTCGCCCAGTT‐3 ^′^.

Actin: forward: 5 ^′^‐CATGTACGTTGCTATCCAGGC‐3 ^′^; reverse: 5 ^′^‐CTCCTTAATGTCACGCACGAT‐3 ^′^.

### 2.11. Clone Formation Assay

Cells were seeded at a density of 1 × 10^3^ per well in a six‐well plate and incubated for 12 days, with medium changes every 3 days. After the culture was completed, 1% paraformaldehyde was added to each well to fix the cells for 30 min, followed by 1 mL crystal violet staining for 20 min, and then waiting to dry before being photographed.

### 2.12. Cell Wound Healing Assay

Six‐well plates were inoculated with 6 × 10^4^ cells per well. At 90% cell density, a sterilized 200 *μ*L pipette tip was used to scratch the well plate, followed by three gentle washes with phosphate‐buffered saline. At this time, the concentration of fetal bovine serum was 1%. The well plate was placed in the 37°C incubator for 24 h. Photographs were taken at 0 and 24 h.

### 2.13. Cell Counting Kit‐8 (CCK‐8) Testing

The CCK‐8 (Cat. RM02823; ABclonal; China) was utilized to assess cell viability. A total of 5 × 10^3^ cells were seeded per well in a 96‐well plate, and a 10‐*μ*L CCK‐8 solution was added to each well after waiting for the cells to adhere and placing them at 37°C for 2 h, and the absorbance values at 450 nm were detected at time points of 0, 24, 48, 72, and 96 h.

### 2.14. Statistical Analysis

The mean and standard deviation were used to express all clinical quantitative indicator values. Independent sample *t*‐tests were utilized to assess the differences between the indicators and the outcomes. A Mann–Whitney *U* test was conducted in all other cases. For the purpose of comparing categorical variables, chi‐square tests were utilized. We employed the log‐rank test to determine the significant differences in the survival rates of the group. The interactions between the expression of ALOX5 and other variables were examined using a likelihood‐ratio test. ALOX5 expression levels were compared across groups with high and low levels using the Wilcoxon test, and the calibration of pathomics‐based predictive models was assessed using the Hosmer–Lemeshow goodness‐of‐fit test. The difference was statistically significant, as shown by the 95% confidence interval (CI) and *p* value of 0.05. Each experiment was repeated three times, and three parallel sample groups were set up each time. We used R software (Version 4.1.0) to perform statistical analyses and ImageJ and GraphPad Prism (Version 8.0.2) software to analyze cell experiments′ images and data. Our study followed the TRIPOD Checklist.

## 3. Results

### 3.1. Patient Characteristics

Following rigorous screening, 327 cases were included in the study. ALOX5 expression levels were separated into high (*n* = 181) and low (*n* = 146) groups based on a cutoff value of 3.527 for ALOX5 expression for the 327 lung cancer patients included in the survival study. Table 1 displays the clinical data for the patients. The differences between distributions of age (*p* = 0.105) and gender (*p* = 0.787) in two ALOX5‐expression groups did not show statistical significance; the pathologic stages were significantly different (*p* = 0.002).

**Table 1:** Patient characteristics of the lung adenocarcinoma cohort.

Variables	Total (*n* = 327)	Low (*n* = 146)	High (*n* = 181)	*p*
Age, *n* (%)				0.105
~65	164 (50)	81 (55)	83 (46)	
66~	163 (50)	65 (45)	98 (54)	
Gender, *n* (%)				0.787
Female	183 (56)	80 (55)	103 (57)	
Male	144 (44)	66 (45)	78 (43)	
Pathologic_stage, *n* (%)				0.002
I/II	265 (81)	107 (73)	158 (87)	
III/IV	62 (19)	39 (27)	23 (13)	
Radiotherapy, *n* (%)				0.008
No	293 (90)	123 (84)	170 (94)	
Yes	34 (10)	23 (16)	11 (6)	
Smoking_status, *n* (%)				0.004
Nonsmoker	41 (13)	12 (8)	29 (16)	
Former	204 (62)	86 (59)	118 (65)	
Current	82 (25)	48 (33)	34 (19)	
Residual_tumor, *n* (%)				0.643
R0	220 (67)	100 (68)	120 (66)	
R1/R2	13 (4)	7 (5)	6 (3)	
RX/unknown	94 (29)	39 (27)	55 (30)	
Histologic_type, *n* (%)				0.775
NOS	204 (62)	94 (64)	110 (61)	
Others	56 (17)	23 (16)	33 (18)	
Mixed subtype	67 (20)	29 (20)	38 (21)	
Tumor_location, *n* (%)				0.677
L‐lower	56 (17)	21 (14)	35 (19)	
L‐upper	76 (23)	33 (23)	43 (24)	
R‐lower	63 (19)	29 (20)	34 (19)	
R‐middle	14 (4)	8 (5)	6 (3)	
R‐upper	118 (36)	55 (38)	63 (35)	
Chemotherapy, *n* (%)				0.137
No	219 (67)	91 (62)	128 (71)	
Yer	108 (33)	55 (38)	53 (29)	

Abbreviation: NOS, not otherwise specified.

### 3.2. High‐ALOX5 Expression Is a Significant Protective Factor for OS in LUAD

This study revealed significantly elevated ALOX5 expression in normal tissues compared to malignant ones (*p* < 0.001; Figure 2a). During the follow‐up process, the low‐ and high‐ALOX5 expression groups′ median survival durations were 41.0 and 53.3 months, respectively. High‐ALOX5 expression levels were linked to OS modifications (*p* = 0.012; Figure 2b). High levels of ALOX5 expression were discovered by univariate analysis to be a determinant in patients′ odds of survival (HR = 0.625; 95% CI, 0.431–0.906; *p* = 0.013). After multiple factor adjustments, a high‐ALOX5 expression level (HR = 0.575; 95% CI, 0.384–0.861; *p* = 0.007) was still a protective factor against OS (Figure 2c). Subgroup analysis revealed that in subgroups of patients aged ≤ 65 years, elevated ALOX5 level was associated with improved OS (HR = 0.501; 95% CI, 0.293–0.856; *p* = 0.011). In addition, the ALOX5 level did not show any significant interactions with the subgroups, with or without radiotherapy or chemotherapy (Figure S2A).

Figure 2Analyses of gene expression and prognosis. (a) Analysis of intergroup differences in ALOX5 levels. (b) Survival analysis of groups showing different levels of ALOX5 expression. (c) Cox regression analysis of clinical features.  ^∗∗∗^ *p* < 0.001. OS, overall survival; NOS, not otherwise specified; 95% CI, 95% confidence interval. Covariates: ALOX5, age, gender, pathologic_stage, radiotherapy, smoking_status, residual_tumor, histologic_type, tumor_location, and chemotherapy.

**Figure:**
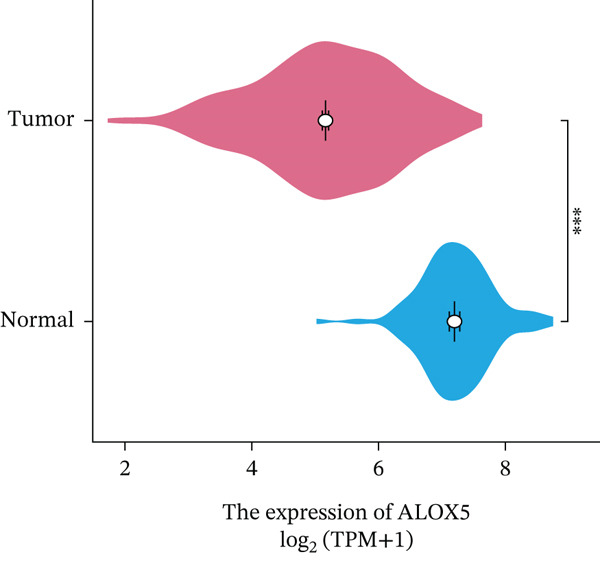
(a)

**Figure:**
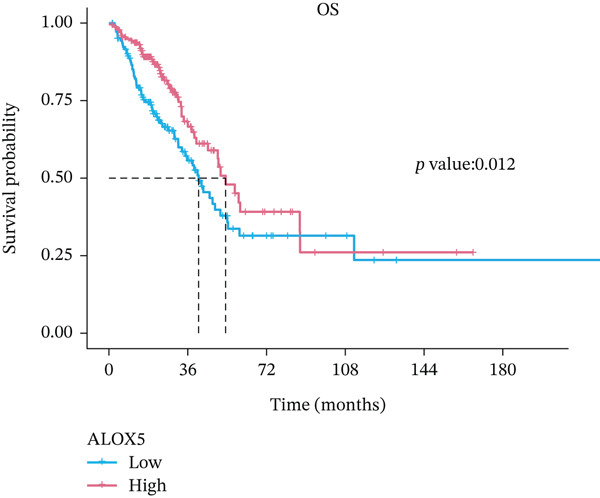
(b)

**Figure:**
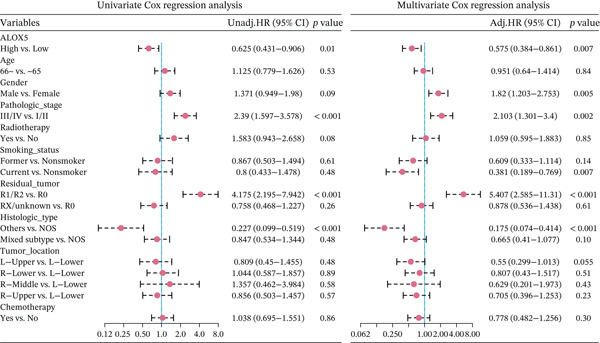
(c)

### 3.3. Screening of Pathological Features

The pathological image feature extraction process is shown in Figure S1. A total of 1488 features, encompassing first‐, second‐, and higher order characteristics, were extracted. The 327 patients in the study were divided into a training set of 230 individuals and a validation set of 97 individuals. Difference analysis of each variable among the groups showed comparability between the groups (Table S1). Once features with zero variance and high correlations (Pearson^′^s correlation coefficient > 0.9) were eliminated, the mRMR method was used to select the Top 30 features, and seven features were obtained by RFE screening (Figure 3a).

Figure 3Pathological feature screening and model establishment and evaluation. (a) The method of screening for pathological features. (b) The importance of the seven features selected. (c) The ROC curve for the training set. (d) The Hosmer–Lemeshow goodness‐of‐fit test for the calibration curve obtained for the training set. (e) The DCA for the training set. (f) The ROC curve for the validation set. (g) Calibration curve and Hosmer–Lemeshow goodness‐of‐fit test for the validation set. (h) The DCA curve for the validation set. ROC, receiver operating characteristic; AUC, area under the curve; DCA, decision curve analysis.

**Figure:**
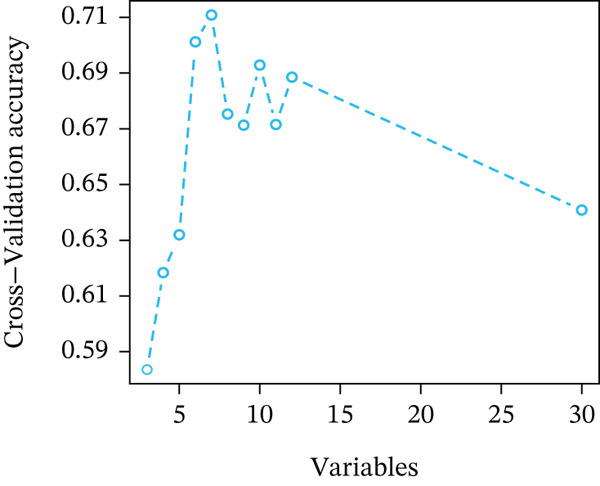
(a)

**Figure:**
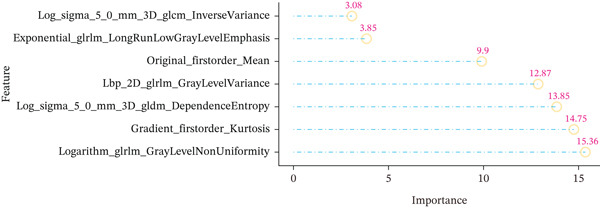
(b)

**Figure:**
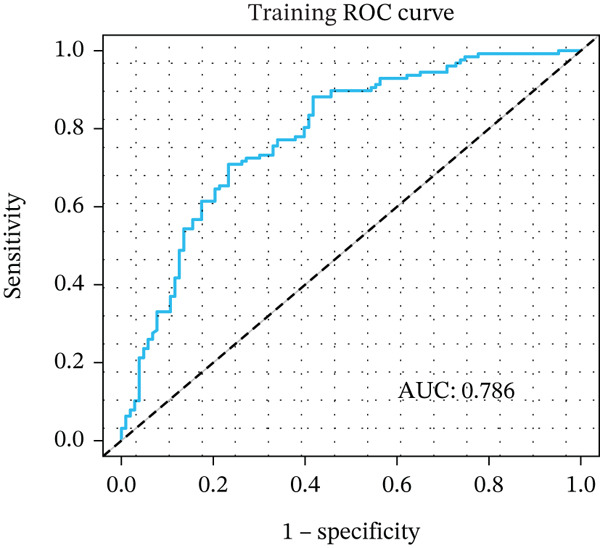
(c)

**Figure:**
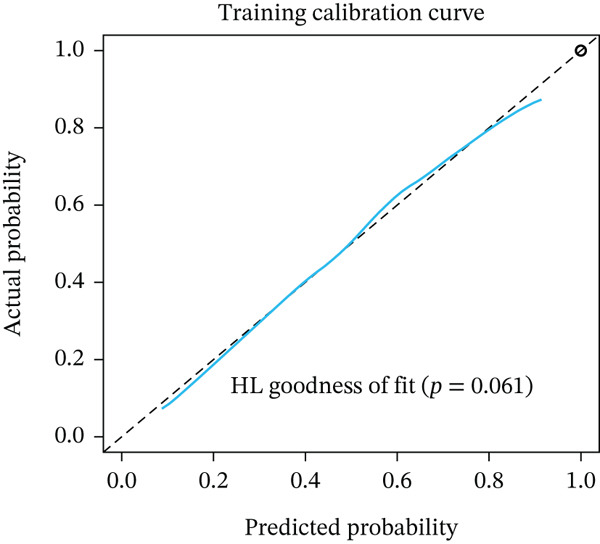
(d)

**Figure:**
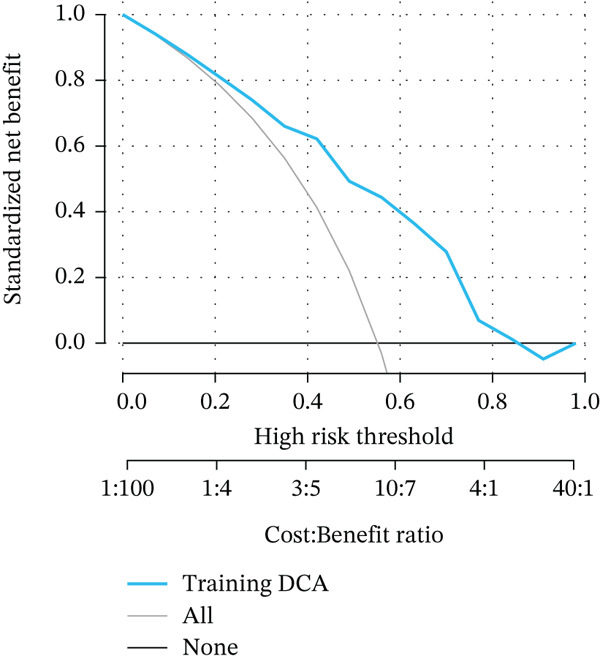
(e)

**Figure:**
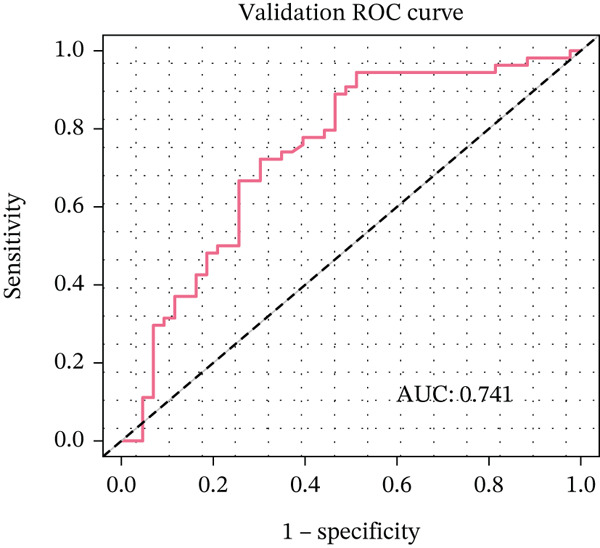
(f)

**Figure:**
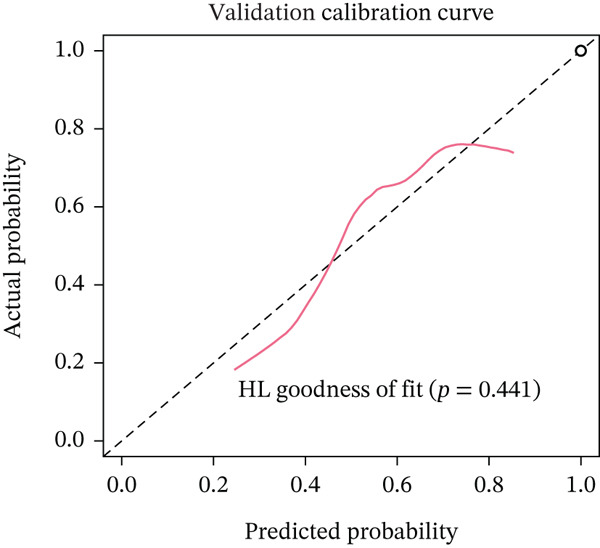
(g)

**Figure:**
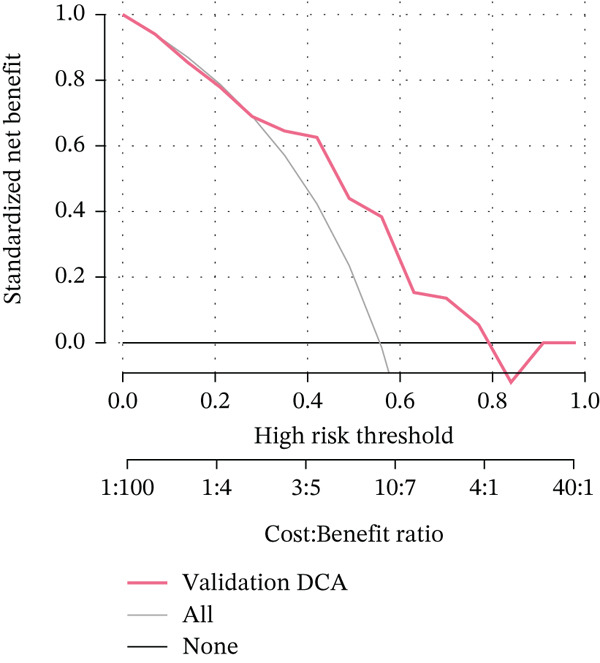
(h)

### 3.4. Building and Assessing Pathomics‐Based Model

Utilizing the GBM technique, we built the model in the training set, and the significance of the seven chosen features is shown in Figure 3b. The receiver operating characteristic (ROC) curve indicated that the model′s area under the curve (AUC) was 0.786 for the training set (Figure 3c) and 0.741 for the validation set (Figure 3f). The pathomics‐based predictive model demonstrated consistency with the predicted gene expression probabilities, as evidenced by the Hosmer–Lemeshow goodness‐of‐fit test and calibration curve (Figure 3d,g). The DCA demonstrated the model′s robust clinical utility and effective predictive performance (Figure 3e,h). Table S2 presents the comprehensive evaluation indicators for the training and validation sets.

### 3.5. High PS Significantly Contributed to Improved OS in LUAD Patients

In the training set, pathomics‐based models showed a significant difference in predicting gene expression levels across various ALOX5 groups (*p* < 0.001). This was validated by consistent results in the validation set, where the high‐ALOX5 expression group exhibited a high PS (Figure 4a). The cutoff value of the PS value predicted by the GBM model was calculated to be 0.514. High and low PS groups were created for the patients (*n* = 197 and 130, respectively; Table 2). For the high PS group, the median survival duration was 51.03 months, whereas for the low PS group, it was 39.9 months, according to the results of the survival analysis. A significant correlation was observed between high PS and improved OS (*p* = 0.003; Figure 4b). Univariate Cox regression analysis identified high PS as a significant protective factor for OS (HR = 0.579; 95% CI, 0.401–0.837; *p* = 0.004). The results were similar after multivariate adjustment (HR = 0.612; 95% CI, 0.411–0.91; *p* = 0.02) (Figure 4c).

Figure 4Prognostic analysis of PS‐based grouping. (a) Differences in PS between high and low PS groups for the training and validation sets. (b) Kaplan–Meier curves for the high and low PS groups. (c) Cox logistic regression analysis. Significance:  ^∗∗∗∗^ *p* < 0.0001. PS, pathomics score. Covariates: PS, age, gender, pathologic_stage, radiotherapy, smoking_status, residual_tumor, histologic_type, tumor_location, and chemotherapy.

**Figure:**
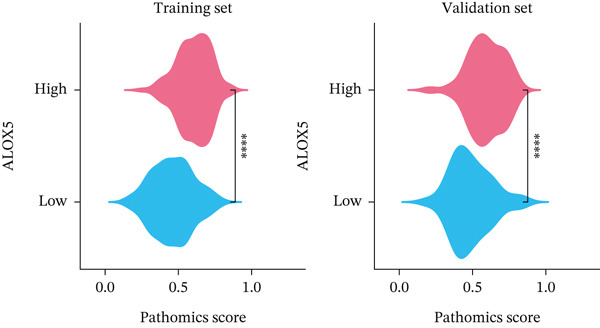
(a)

**Figure:**
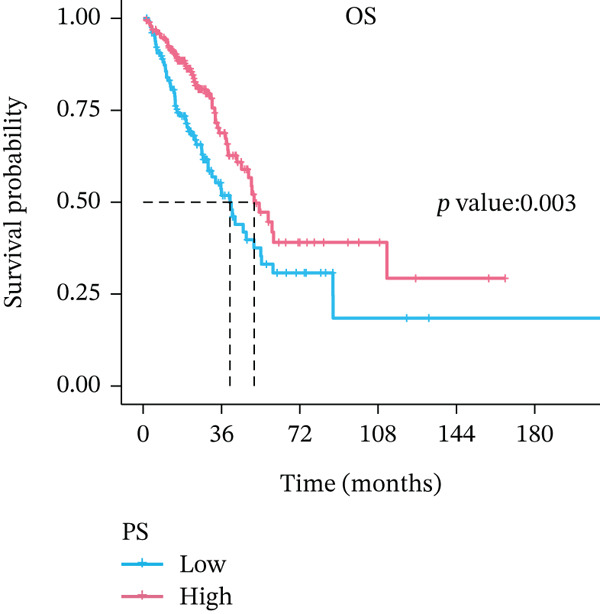
(b)

**Figure:**
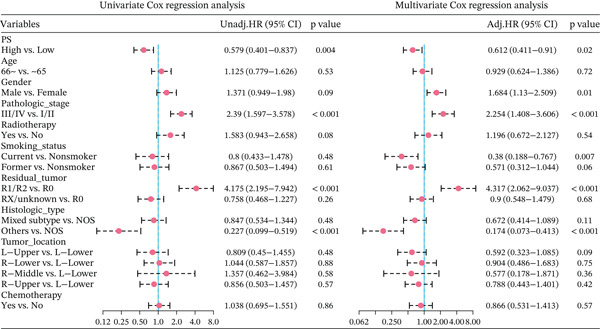
(c)

**Table 2:** Clinical characteristics of different PS groups.

Variables	Total (*n* = 327)	High (*n* = 197)	Low (*n* = 130)	*p*
Age, *n* (%)				0.769
~65	164 (50)	97 (49)	67 (52)	
66~	163 (50)	100 (51)	63 (48)	
Gender, *n* (%)				0.459
Female	183 (56)	114 (58)	69 (53)	
Male	144 (44)	83 (42)	61 (47)	
Pathologic_stage, *n* (%)				0.092
I/II	265 (81)	166 (84)	99 (76)	
III/IV	62 (19)	31 (16)	31 (24)	
Radiotherapy, *n* (%)				1
No	293 (90)	177 (90)	116 (89)	
Yes	34 (10)	20 (10)	14 (11)	
Smoking_status, *n* (%)				0.145
Nonsmoker	41 (13)	30 (15)	11 (8)	
Current	82 (25)	45 (23)	37 (28)	
Former	204 (62)	122 (62)	82 (63)	
Residual_tumor, *n* (%)				0.558
R0	220 (67)	133 (68)	87 (67)	
R1/R2	13 (4)	6 (3)	7 (5)	
RX/unknown	94 (29)	58 (29)	36 (28)	
Histologic_type, *n* (%)				0.773
NOS	204 (62)	122 (62)	82 (63)	
Mixed subtype	67 (20)	39 (20)	28 (22)	
Others	56 (17)	36 (18)	20 (15)	
Tumor_location, *n* (%)				0.034
L‐lower	56 (17)	27 (14)	29 (22)	
L‐upper	76 (23)	42 (21)	34 (26)	
R‐lower	63 (19)	40 (20)	23 (18)	
R‐middle	14 (4)	6 (3)	8 (6)	
R‐upper	118 (36)	82 (42)	36 (28)	
Chemotherapy, *n* (%)				0.287
No	219 (67)	127 (64)	92 (71)	
Yes	108 (33)	70 (36)	38 (29)	

Abbreviation: PS, pathomics score.

### 3.6. Analysis of Immune Cell Abundance and Signaling Pathways

According to the immune cell infiltration in each sample, as seen in Figure 5a, the high PS group had a higher abundance of M0, M1, and M2 macrophages (*p* < 0.05). We have supplemented Table S3 to show the *p* values of immune cell abundance during immune cell deconvolution. GSEA of the KEGG gene set highlighted the Top 20 pathways, with differentially expressed genes in the PS high and low groups significantly enriched in the MAPK signaling pathway (Figure S2B). Regarding the Hallmark gene set, apoptosis and the inflammatory response signaling pathways were highly enriched in these two groups (Figure 5b). Further investigation into the relationship between PS and apoptotic gene expression showed a substantial positive connection between the PS and the expression of apoptotic genes, including *PIK3R5, FAS*, and *PIK3CG* (*p* < 0.001) (Figure 5c). The optimal soft threshold power, based on scale‐free networks, was eight (Figure S2C). Two modules were subsequently designed using average connected hierarchical clustering and the optimal soft threshold power. We assigned 199 genes to the two modules. The brown module was closely linked to LUAD patients′ prognosis based on the Pearson correlation coefficient value for the sample features of each module. Therefore, the brown module was determined to be a key module (Figure 5d). GO enrichment analysis revealed significant enrichment of 63 hub genes in the brown module within cytokine‐mediated signaling pathways (Figure 5e). KEGG analysis indicated a significant overrepresentation of the 63 hub genes in the brown module within the Toll‐like receptor signaling pathway (Figure S2D).

Figure 5Analysis of pathological mechanisms. (a) The expression of immune cell abundance between high and low PS groups. (b) Perform GSEA on the Hallmark gene set. (c) The correlation between PS and the expression of apoptosis genes. (d) Use WGCNA to search for a set of inflammatory response genes related to prognosis. (e) GO enrichment analysis of hub genes related to inflammatory response. ns, no significance.  ^∗^ *p* < 0.05,  ^∗∗^ *p* < 0.005, and  ^∗∗∗^ *p* < 0.0005. GSEA, gene set enrichment analysis; WGCNA, weighted gene coexpression network analysis; GO, Gene Ontology; MF, molecular function; BP, biological process; CC, cellular component.

**Figure:**
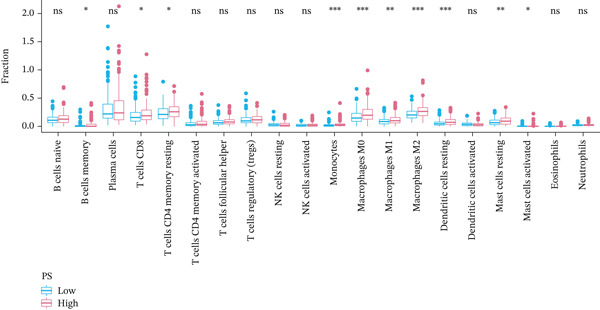
(a)

**Figure:**
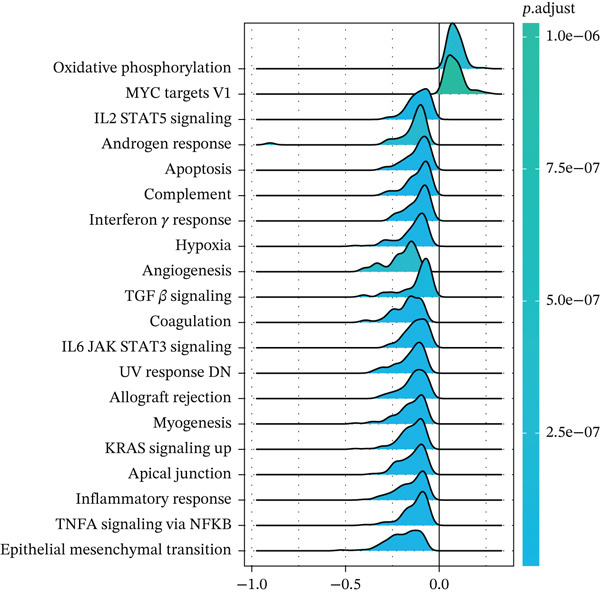
(b)

**Figure:**
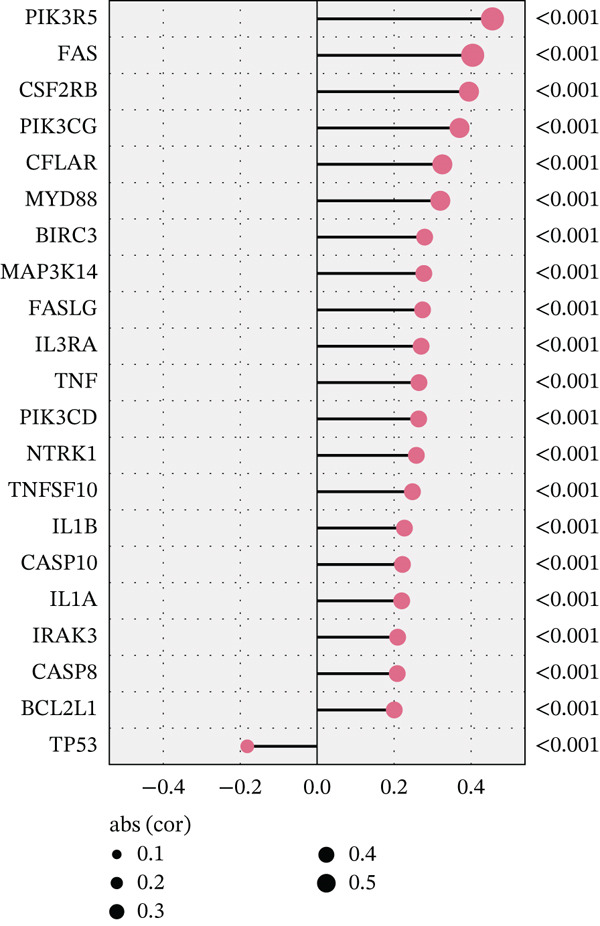
(c)

**Figure:**
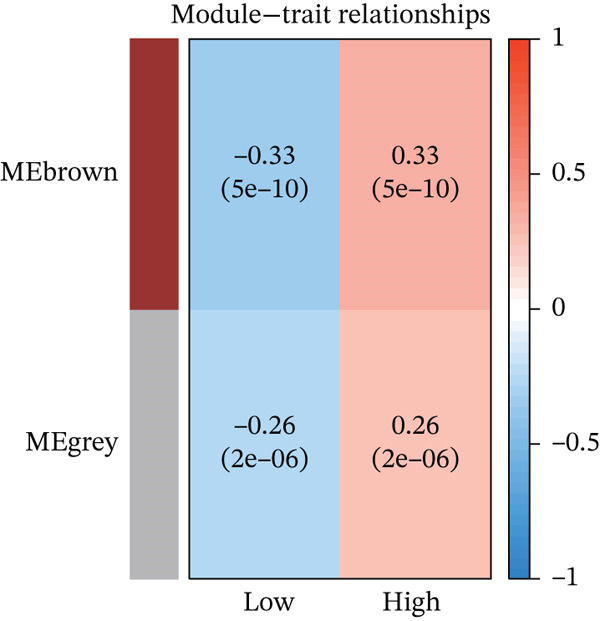
(d)

**Figure:**
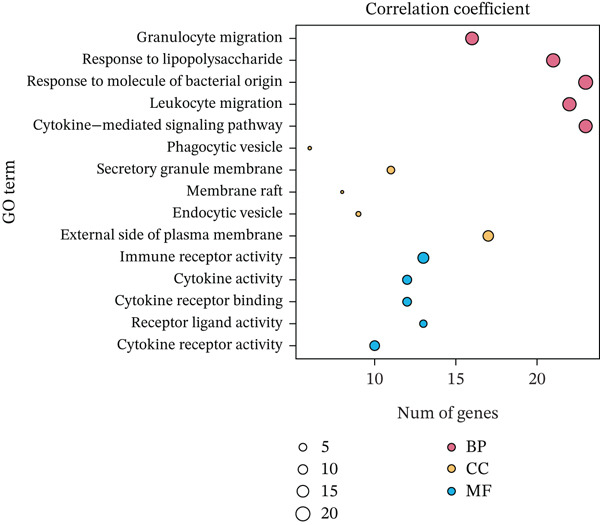
(e)

### 3.7. ALOX5 Enhances Cellular Proliferation and Migration

To investigate the functional role of ALOX5 in lung cancer cells, we conducted a series of cellular assays. Initial WB (Figure 6a) and qRT‐PCR (Figure 6b) analyses revealed differential ALOX5 expression across HBE and multiple lung cancer cell lines (PC9, H1975, H1299, HCC827, H23, H460, A549, and H2170). Based on expression profiles, we selected two high‐expressing (A549 and PC9) and two low‐expressing (H1975 and H23) LUAD cell lines for subsequent knockdown and overexpression experiments. Validation of genetic manipulation efficacy was performed through WB (knockdown: Figure 6c,d; overexpression: Figure 6e,f) and qRT‐PCR (knockdown: Figure 6g,h; overexpression: Figure 6i,j). Two optimal knockdown cell lines from A549 and PC9 were selected for phenotypic characterization. CCK‐8 assays demonstrated enhanced proliferation in ALOX5‐knockdown PC9 (Figure 6k) and A549 cells (Figure S3A), whereas overexpression attenuated proliferative capacity in H23 (Figure 6k) and H1975 cells (Figure S3B). Colony formation assays corroborated these findings, showing enhanced clonogenic potential in ALOX5‐knockdown PC9 (Figure 6l) and A549 cells (Figure S3C). However, H23 (Figure 6m) and H1975 (Figure S3D) showed opposite results. Migration analysis revealed significantly elevated 24‐h scratch wound healing rates in ALOX5‐deficient PC9 (Figure 6n) and A549 cells (Figure S3E). Conversely, ALOX5‐overexpressing H23 (Figure 6o) and H1975 cells (Figure S3F) exhibited markedly impaired migratory capacity compared to controls.

Figure 6Functional validation of gene expression through cellular experiments. (a) Western blot (WB) analysis of ALOX5 expression across various lung cancer cell lines. (b) Quantitative real‐time polymerase chain reaction (qRT‐PCR) assay assessing ALOX5 expression levels in distinct lung cancer cell lines. (c) Verification of ALOX5 knockdown in A549 cells via WB. (d) Confirmation of ALOX5 knockdown in PC9 cells using WB. (e) Validation of ALOX5 overexpression in H1975 cells by WB. (f) WB demonstrating ALOX5 overexpression in H23 cells. (g) qRT‐PCR‐based confirmation of ALOX5 knockdown in A549 cells. (h) qRT‐PCR analysis verifying ALOX5 knockdown in PC9 cells. (i) qRT‐PCR validation of ALOX5 overexpression in H1975 cells. (j) qRT‐PCR assay confirming ALOX5 overexpression in H23 cells. (k) Cell counting kit‐8 (CCK‐8) assay evaluating proliferation in PC9 ALOX5‐knockdown and H23 ALOX5‐overexpressing cells. (l) Colony formation assay of PC9 ALOX5‐knockdown cell lines. (m) Colony formation assay of H23 ALOX5‐overexpressing cell lines. (n) Scratch wound healing assay in PC9 ALOX5‐knockdown cells. (o) Scratch wound healing assay in H23 ALOX5‐overexpressing cells.  ^∗^ *p* < 0.05,  ^∗∗^ *p* < 0.005, and  ^∗∗∗^ *p* < 0.0005.

**Figure:**
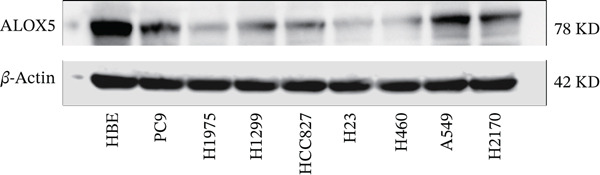
(a)

**Figure:**
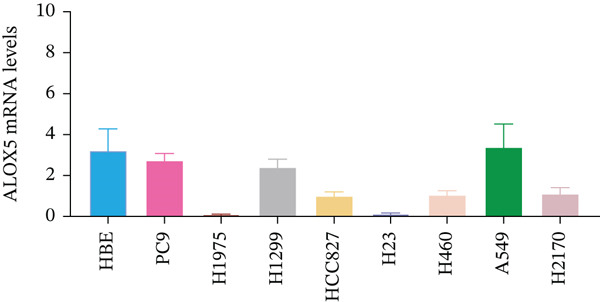
(b)

**Figure:**
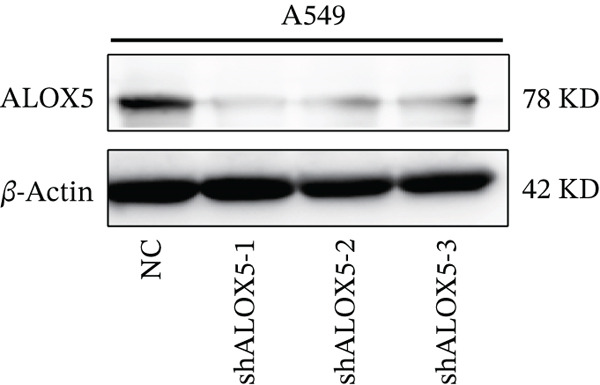
(c)

**Figure:**
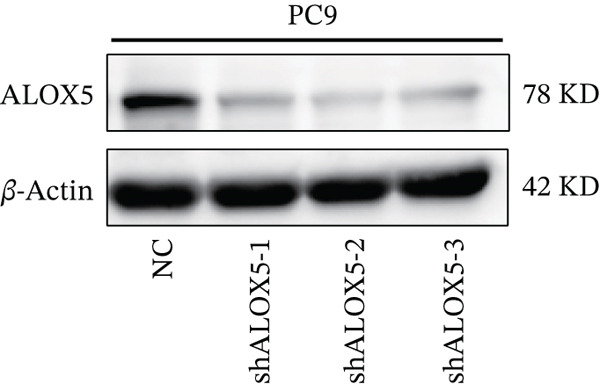
(d)

**Figure:**
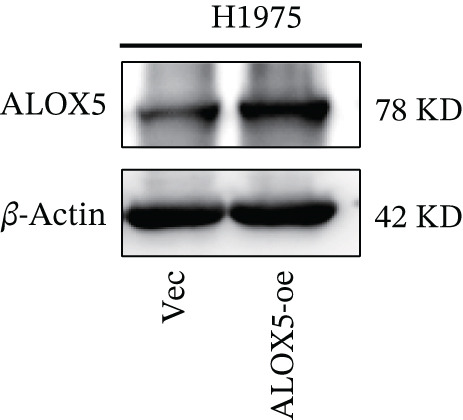
(e)

**Figure:**
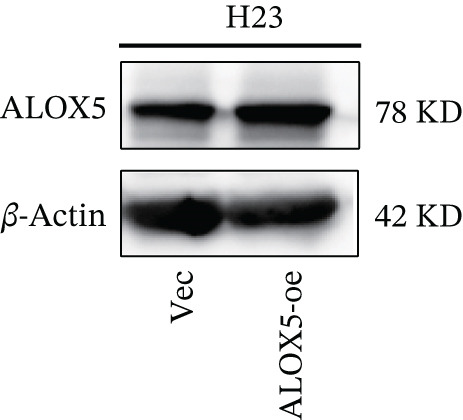
(f)

**Figure:**
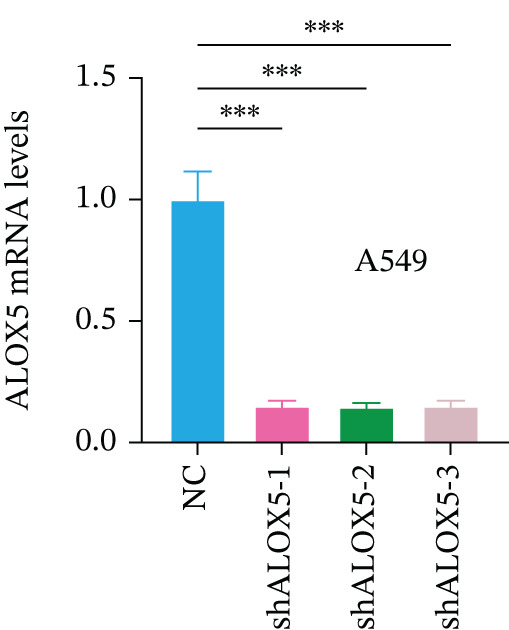
(g)

**Figure:**
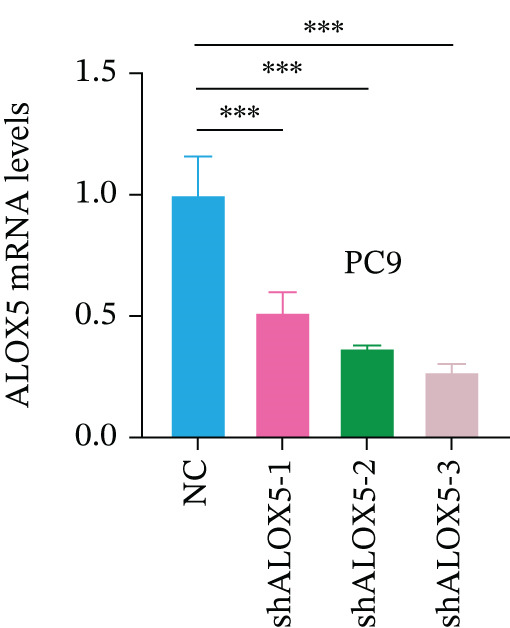
(h)

**Figure:**
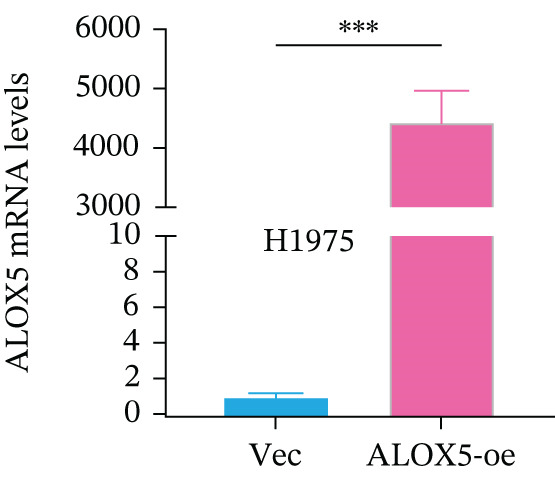
(i)

**Figure:**
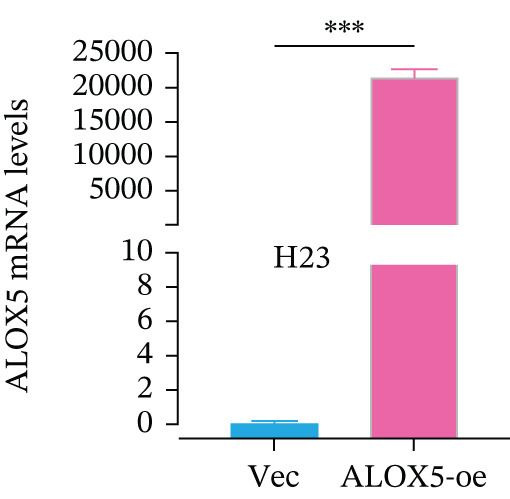
(j)

**Figure:**
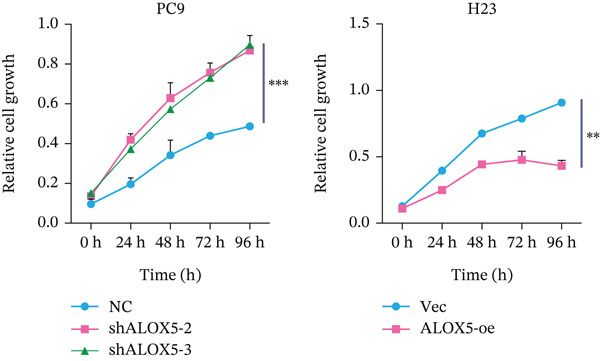
(k)

**Figure:**
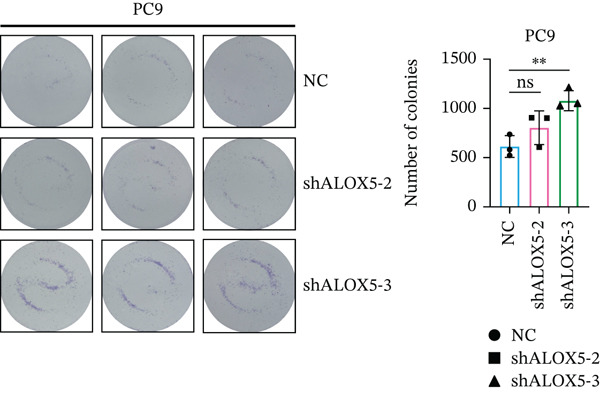
(l)

**Figure:**
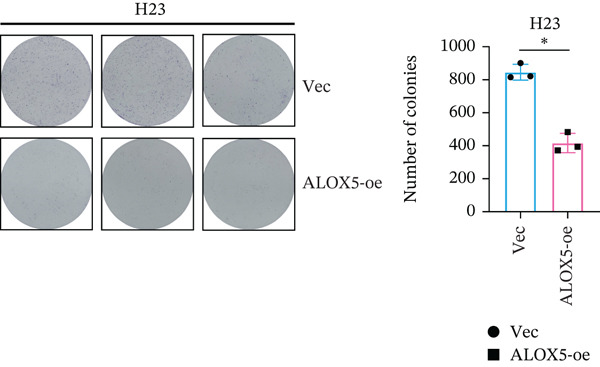
(m)

**Figure:**
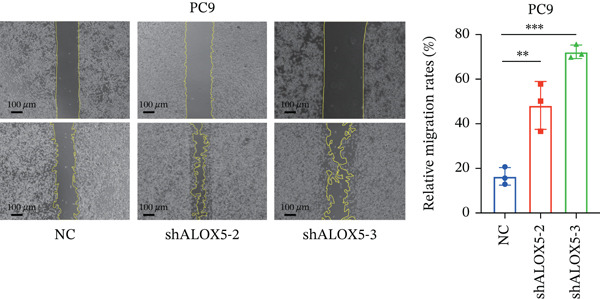
(n)

**Figure:**
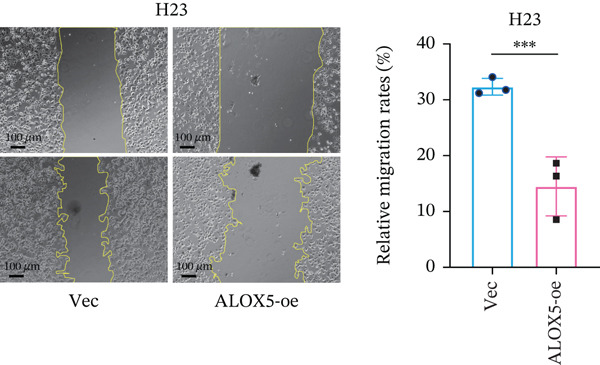
(o)

## 4. Discussion

This is the first publication that uses histopathological imaging features to forecast the level of ALOX5 expression. We observed that patients with lung cancer had a better prognosis the more ALOX5 was expressed. We extracted histopathological imaging features, established a prognostic model using machine learning algorithms for the training set, and confirmed the model′s predictive effectiveness using the validation set. In addition, the effects of ALOX5 gene knockout and overexpression on cell proliferation, colony formation, and cell migration ability were verified by cell phenotype experiments. Our results showed that ALOX5 levels can predict the OS of patients with lung adenocarcinoma, and the histopathological feature–based machine learning model can reliably estimate the expression status of ALOX5, thereby predicting patient prognosis. An effective approach for forecasting the prognosis of lung adenocarcinoma patients in clinical practice may be the integration of histological pictures and ALOX5 levels in the pathomics‐based model.

Based on our analysis, we believe that a high expression level of ALOX5 indicated better OS, and subsequent pathway analysis revealed that this may be because ALOX5 mediates inflammation and triggers apoptosis. In previous studies on other types of cancers, ALOX5 levels were found to be closely associated with prognosis. Zhang et al. [15] observed that patients having colon cancer with elevated ALOX5 expression levels had a poor outcome; Liu et al. [16] observed that bladder cancer patients with high expression levels of ALOX5 had a good prognosis. The variation in levels of ALOX5 expression and prognosis in different cancer types may be due to differences in genes and phenotypes among different tumor cells.

Artificial intelligence has a broad application prospect in disease diagnosis. A histological analysis of the postoperative tissues from 152 non–small cell lung cancer sufferers by Kiliçgün et al. [17] revealed that both the presence of necrosis and perineural invasion independently suggested a poor outcome. After postoperatively analyzing the pathological features of 247 small cell lung cancer sufferers, Liu et al. [18] concluded that spindle cell–type and tumor‐infiltrating lymphocytes (TILs; > 30%) were rejuvenated as separate adverse diagnostic factors. The advantage of our model is that we included a large number of 327 patients, and because of which, the results are convincing. In our study, we extracted pathological features of 1488 tissue samples and used mRMR and RFE to screen seven features. These seven features comprised three gray‐level run‐length matrices (GLRLMs), one gray‐level dependence matrix (GLDM), and two first‐order features. The results indicate that second‐order texture features were related to survival outcomes. In contrast to the findings of previous studies, by integrating histopathological image features with transcriptomic data through multimodal data analysis, we have expanded the methodological framework for disease prognosis prediction. The H&E staining technique is simple, easy to perform, and has a low error rate and low cost. We can use H&E pathological sections to predict the expression of ALOX5, which can avoid the error caused by the operation error in the process of IHC and can also reduce the cost of the antibody, which has good practical value in clinical practice. Overall, analysis of pathological imaging features can be a convenient and low‐cost method for predicting ALOX5 expression levels in patients with LUAD. Different from traditional pathological diagnosis, machine learning of the morphological characteristics of pathological sections, combined with clinical data, transcriptome data, and other multiomics data of patients, can help doctors make better clinical diagnoses and guide clinical decision‐making.

On a mechanistic level, usually, M1 and M2 macrophages determine the fate of organs during inflammation, which indicates that future studies on functional annotation should focus on inflammatory response–related pathways. Previous studies have shown that macrophages regulate iron metabolism and iron homeostasis [19]. ALOX5 plays a crucial role in iron‐mediated death [20]. A potential regulatory relationship between tumor immunity and iron‐mediated death has been previously reported [21]. We conducted GSEA using KEGG and Hallmark gene sets and identified enrichment in the MAPK signaling pathway, cell apoptosis, and inflammatory response. Prior research indicates that lipid peroxidation products create adducts with *ERK*, *JNK*, and *p38*, thereby activating MAPKs and caspase signaling to trigger apoptosis‐related processes [22–24]. Yu et al. [25] concluded that iron‐mediated death was closely related to inflammatory responses. The findings of prior research are congruent with ours, providing strong evidence that ferroptosis is involved in multiple biological pathways in tumors and has a significant impact on the growth of tumors.

We acknowledge that multiple clinical and technical variables may simultaneously affect pathomics features and prognostic outcomes, thereby introducing confounding. In this study, the main potential confounders considered included age, gender, smoking history, TNM stage, and histologic subtype. If not properly controlled, these factors may lead to misinterpretation of the biological significance. To address this, we reported and compared the distributions of key clinical variables in the baseline table and performed outcome analyses using multivariable Cox regression, including age, gender, smoking history, TNM stage, and histologic subtype as covariates to evaluate the independent predictive value of the PS. After multivariable adjustment, PS remained an independent predictor (adjusted HR = 0.612, 95% CI 0.411–0.910, *p* = 0.015), supporting the robustness of our findings. However, despite these measures, several important limitations remain—such as residual confounding, sampling variation, and batch differences. Future work should prioritize multicenter, prospective cohort studies with standardized specimen processing and scanning to obtain more representative samples and enable independent external validation and should systematically collect more comprehensive clinical and molecular annotations (including detailed treatment histories, comorbidity scores, and expanded molecular markers) to better control confounding and investigate potential interactions.

Our study faced certain limitations. One limitation is that we obtained data from public databases, and the data were not validated using external datasets. We are currently establishing a multicenter prospective cohort with standardized specimen processing and scanning protocols, and we anticipate that a high‐quality external validation cohort will be available for inclusion in this study in the near future. Second, we included cases of patients with LUAD, and no other subtypes of cases with other types of cancers were included. Third, choosing representative subimages through pathological examination of histological images may introduce selection bias. Our study found that high‐ALOX5 expression is associated with improved prognosis, but its molecular mechanisms have not yet been systematically validated. Based on the literature and our observations, we propose several testable mechanistic hypotheses; for example, ALOX5 may enhance antitumor immunity by modulating the tumor microenvironment, or its high expression may reflect a favorable cellular composition or a less invasive differentiated state; these possibilities remain speculative. We plan to prioritize mechanistic studies in future multicenter collaborations and prospective cohorts and will report supporting mechanistic data in subsequent publications once available. Despite these limitations, our prognostic model showed satisfactory performance, providing a new approach for clinical decision‐making.

## 5. Conclusion

ALOX5 expression is significantly associated with lung adenocarcinoma prognosis and may act as a prognostic molecular marker. A predictive model utilizing pathological features serves as an effective tool for aiding clinical decisions and guiding personalized, precise diagnosis and treatment.

## Author Contributions

Peihong Hu and Chun Huang contributed equally to this work as first authors.

## Funding

This study was funded by the Sichuan Medical Association Research Project (No. 2024HR13).

## Ethics Statement

The patient data used in this study were obtained from public databases and were exempt from ethical review.

## Conflicts of Interest

The authors declare no conflicts of interest.

## Supporting information


**Supporting Information** Additional supporting information can be found online in the Supporting Information section. The 327 patients in the study were divided into a training set of 230 individuals and a validation set of 97 individuals. Table S1 shows the analysis of differences in various variables among the groups. Table S2 presents the comprehensive evaluation indicators for the training and validation sets. Table S3 shows the *p* values for immune cell abundances obtained from immune cell deconvolution. The pathological image feature extraction process is shown in Figure S1. Figure S2 shows the impact of high‐ versus low‐ALOX5 expression on patient prognosis across subgroups stratified by various covariates, along with GSEA of KEGG gene sets, WGCNA, and KEGG enrichment analysis. Figure S3 shows the phenotypic assays of A549 and H1975 cells.

## Data Availability

The data that support the findings of this study are available from the corresponding authors upon reasonable request.
