# Supplementary material for: ALOX5 Expression and Pathomics Features Reveal New Insights Into Lung Adenocarcinoma Prognosis: Model Construction and Functional Validation
Source: Hum Mutat. 2026 Mar 9;2026:3303601. doi: 10.1155/humu/3303601 (PMC12968730; doi:10.1155/humu/3303601)
Supplement: Supplementary file 1 — Supporting Information Additional supporting information can be found online in the Supporting Information section. The 327 patients in the study were divided into a training set of 230 individuals and a validation set of 97 individuals. Table S1 shows the analysis of differences in various variables among the groups. Table S2 presents the comprehensive evaluation indicators for the training and validation sets. Table S3 shows the p values for immune cell abundances obtained from immune cell deconvolution. The pathological image feature extraction process is shown in Figure S1. Figure S2 shows the impact of high‐ versus low‐ALOX5 expression on patient prognosis across subgroups stratified by various covariates, along with GSEA of KEGG gene sets, WGCNA, and KEGG enrichment analysis. Figure S3 shows the phenotypic assays of A549 and H1975 cells. [file HUMU-2026-3303601-s001.docx]

**Supplementary material**

**Table S1** Partitioning of data sets.

| Variables | Total (n = 327) | Train (n = 230) | Validation (n = 97) | *P* |
| --- | --- | --- | --- | --- |
| ALOX5, n (%) |  |  |  | 1 |
| Low | 146 (45) | 103 (45) | 43 (44) |  |
| High | 181 (55) | 127 (55) | 54 (56) |  |
| Age, n (%) |  |  |  | 0.603 |
| ~65 | 164 (50) | 118 (51) | 46 (47) |  |
| 66~ | 163 (50) | 112 (49) | 51 (53) |  |
| Gender, n (%) |  |  |  | 0.848 |
| Female | 183 (56) | 130 (57) | 53 (55) |  |
| Male | 144 (44) | 100 (43) | 44 (45) |  |
| Pathologic_stage, n (%) |  |  |  | 0.115 |
| I/II | 265 (81) | 192 (83) | 73 (75) |  |
| III/IV | 62 (19) | 38 (17) | 24 (25) |  |
| Radiotherapy, n (%) |  |  |  | 1 |
| NO | 293 (90) | 206 (90) | 87 (90) |  |
| YES | 34 (10) | 24 (10) | 10 (10) |  |
| Residual_tumor, n (%) |  |  |  | 0.506 |
| R0 | 220 (67) | 159 (69) | 61 (63) |  |
| R1/R2 | 13 (4) | 9 (4) | 4 (4) |  |
| RX/Unknown | 94 (29) | 62 (27) | 32 (33) |  |
| Histologic_type, n (%) |  |  |  | 0.359 |
| NOS | 204 (62) | 146 (63) | 58 (60) |  |
| Mixed Subtype | 67 (20) | 49 (21) | 18 (19) |  |
| Others | 56 (17) | 35 (15) | 21 (22) |  |
| Tumor_location, n (%) |  |  |  | 0.876 |
| L-Lower | 56 (17) | 42 (18) | 14 (14) |  |
| L-Upper | 76 (23) | 52 (23) | 24 (25) |  |
| R-Lower | 63 (19) | 43 (19) | 20 (21) |  |
| R-Middle | 14 (4) | 9 (4) | 5 (5) |  |
| R-Upper | 118 (36) | 84 (37) | 34 (35) |  |
| Chemotherapy, n (%) |  |  |  | 0.373 |
| NO | 219 (67) | 158 (69) | 61 (63) |  |
| YES | 108 (33) | 72 (31) | 36 (37) |  |
| OS, n (%) |  |  |  | 0.738 |
| 0 | 213 (65) | 148 (64) | 65 (67) |  |
| 1 | 114 (35) | 82 (36) | 32 (33) |  |
| OS.time, Median (Q1,Q3) | 21.73 (14.48, 35.1) | 22.03 (15.54, 35.95) | 20.1 (11.77, 33.17) | 0.122 |

**Table S2** Comprehensive evaluation index of training set and validation set.

| name | ALOX5_cat_PS | name | ALOX5_cat_PS |
| --- | --- | --- | --- |
| train_auc | 0.786 | test_auc | 0.741 |
| train_ci | 0.726-0.846 | test_ci | 0.638-0.845 |
| Train_thre | 0.551 | Test_acc | 0.691 |
| Train_acc | 0.735 | Test_sens | 0.648 |
| Train_sens | 0.709 | Test_spec | 0.744 |
| Train_spec | 0.767 | Test_ppv | 0.761 |
| Train_ppv | 0.789 | Test_npv | 0.627 |
| Train_npv | 0.681 | test_brierScore | 0.214 |
| train_brierScore | 0.197 |  |  |

**Table S3** Differential analysis of immune cell abundance.

| Variable | *P*_value | Variable | *P*_value |
| --- | --- | --- | --- |
| B cells naive | 0.113540833 | Monocytes | 6.84E-05 |
| B cells memory | 0.032117667 | Macrophages M0 | 0.000839436 |
| Plasma cells | 0.885953158 | Macrophages M1 | 0.005484619 |
| T cells CD8 | 0.032302738 | Macrophages M2 | 6.19E-07 |
| T cells CD4 memory resting | 0.011280834 | Dendritic cells resting | 5.98E-05 |
| T cells CD4 memory activated | 0.187398688 | Dendritic cells activated | 0.149738378 |
| T cells follicular helper | 0.088293358 | Mast cells resting | 0.001569499 |
| T cells regulatory (Tregs) | 0.202624887 | Mast cells activated | 0.036596061 |
| NK cells resting | 0.381672627 | Eosinophils | 0.714421196 |
| NK cells activated | 0.36771493 | Neutrophils | 0.066731597 |

**
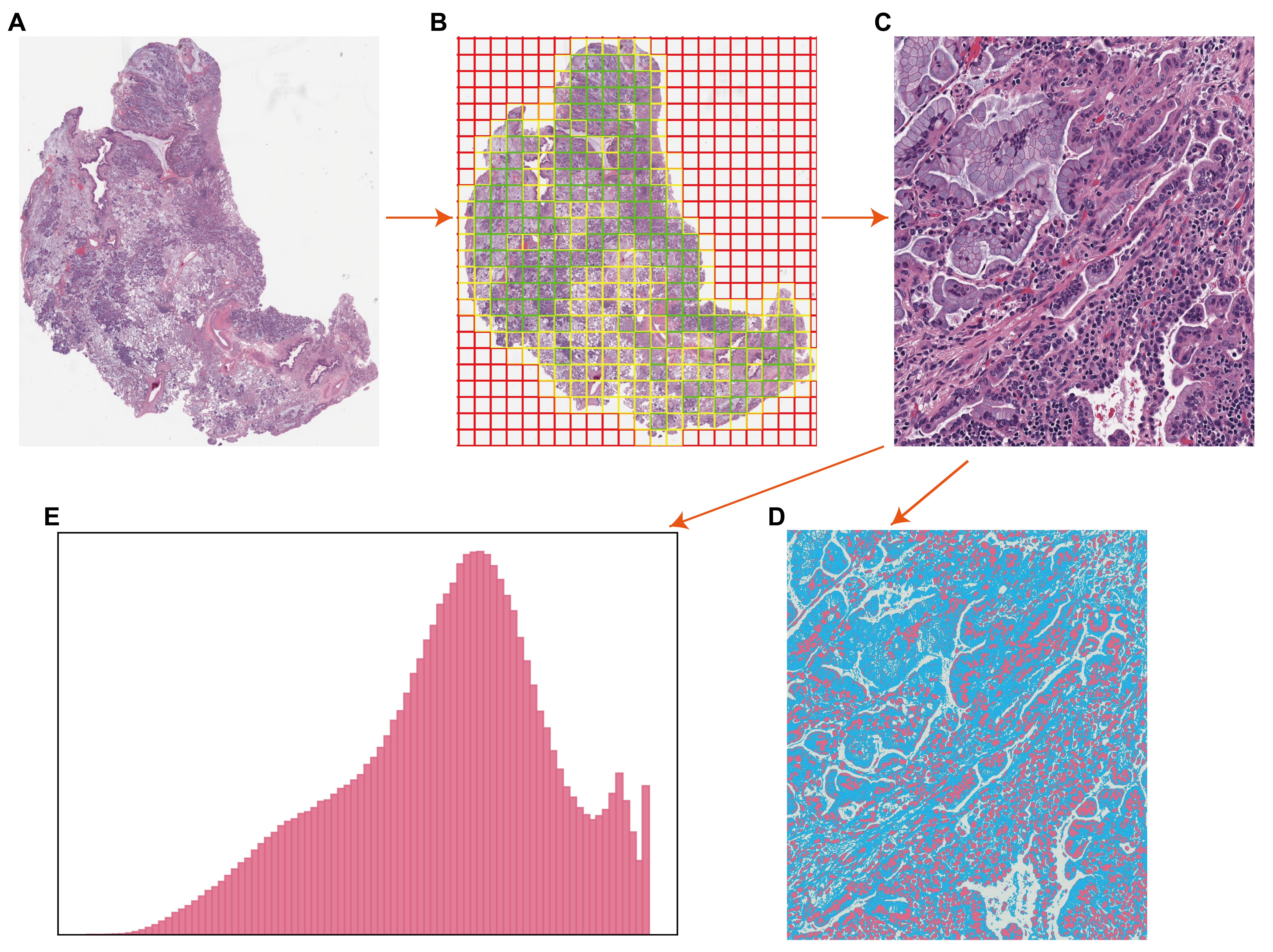
**

**FigureS1. The process of pathological feature extraction. A. IPathological images. B. Pathological image segmentation. C. Pathological subimages. D. Standardization of images. E. Feature extraction.**

**
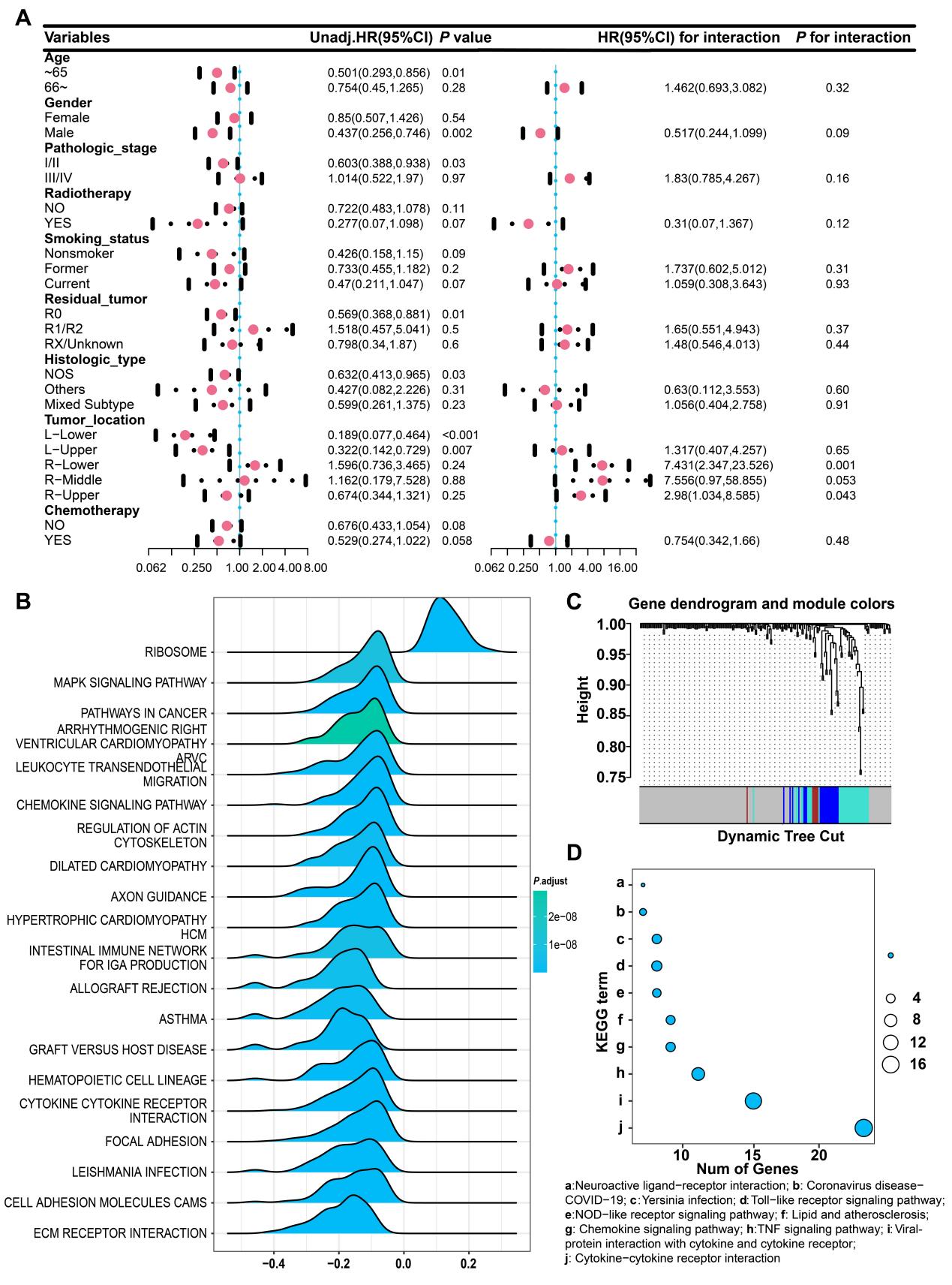
**

**FigureS2. Clinical information and prognosis and Enrichment analysis of related genes. A. Interaction analysis of clinical features. B. GSEA enrichment analysis in KEGG gene collection. C. WGCNA analysis. D. KEGG enrichment analysis.**

**
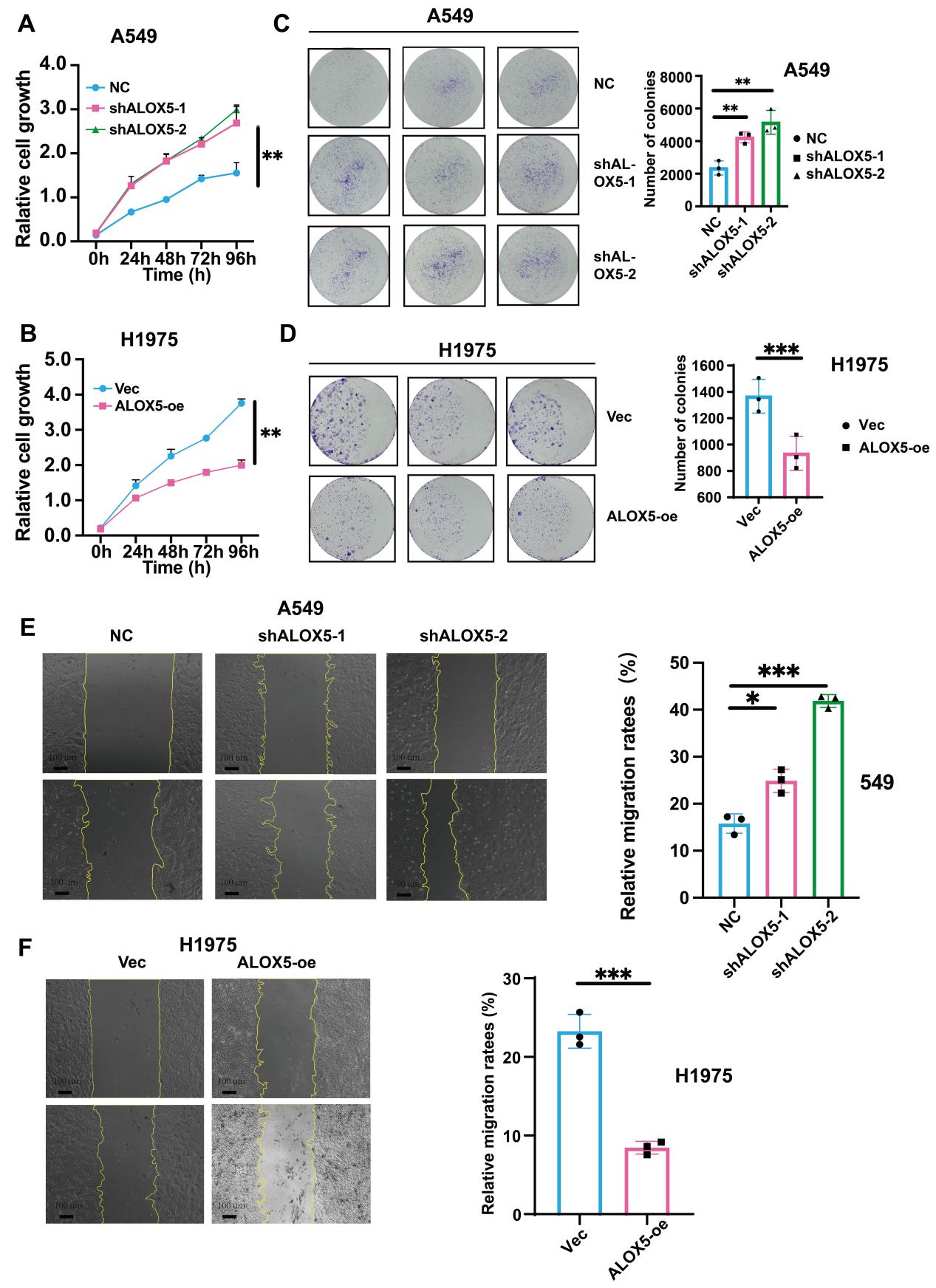
**

**Figgure S3. Cell phenotype experiments. A. CCK-8 assay evaluating proliferation in A549 ALOX5-knockdown cells. B. CCK-8 assay evaluating proliferation in H1975 ALOX5-overexpressing cells. C. Colony formation assay of A549 ALOX5-knockdown cell lines. D. Colony formation assay of H1975 ALOX5-overexpressing cell lines. E. Scratch wound healing assay in A549 ALOX5-knockdown cells. F. Scratch wound healing assay in H1975 ALOX5-overexpressing cells. *, *P* < 0.05, **, *P* < 0.005; ***, *P* < 0.0005.**
